# A plasmid-encoded *papB* paralogue modulates autoaggregation of *Escherichia coli* transconjugants

**DOI:** 10.1186/s13104-020-05405-7

**Published:** 2020-12-14

**Authors:** Rubén Monárrez, Iruka N. Okeke

**Affiliations:** 1 Department of Biology, Haverford College, Haverford, Pennsylvania USA; 2 Department of Pharmaceutical Microbiology, Faculty of Pharmacy, University of Ibadan, Ibadan, Oyo State Nigeria

**Keywords:** Plasmid, PapB, PefB, Fimbrial regulation, Autoaggregation

## Abstract

**Objective:**

Plasmids are key to antimicrobial resistance transmission among enteric bacteria. It is becoming increasingly clear that resistance genes alone do not account for the selective advantage of plasmids and bacterial strains that harbor them. Deletion of a 32 Kb fitness-conferring region of pMB2, a conjugative resistance plasmid, produced a hyper-autoaggregation phenotype in laboratory *Escherichia coli*. This study sought to determine the genetic basis for hyper-autoaggregation conferred by the pMB2-derived mini-plasmid.

**Results:**

The 32 Kb fragment deleted from pMB2 included previously characterized nutrient acquisition genes as well as putative transposase and integrase genes, a 272 bp *papB/ pefB*-like gene, and several open-reading frames of unknown function. We cloned the *papB/ pefB* paralogue and found it sufficient to temper the hyper-autoaggregation phenotype. Hyper-autoaggregation conferred by the mini-plasmid did not occur in a *fim*-negative background. This study has identified and characterized a gene capable of down-regulating host adhesins and has shown that trans-acting *papB/pefB* paralogues can occur outside the context of an adhesin cluster. This plasmid-mediated modification of a bacterial host’s colonization program may optimize horizontal transfer of the mobile element bearing the genes.

## Introduction

Bacterial autoaggregation is an adherence phenotype that can manifest in macroscopical clumping. Autoaggregation enhances colonization of some niches but because it is not always advantageous, it is typically regulated transcriptionally or post-transcriptionally by antiaggregation proteins or steric hindrance [1–5]. The *Escherichia coli* pangenome contains hundreds of known autoaggregation/antiaggregation factors, many of which also function in adherence to other cells and surfaces and have been studied in the context of specific virulent *E. coli* strains or pathotypes [4].

We recently isolated and sequenced a large multi-drug resistance plasmid from a commensal *Escherichia* strain in Nigeria and mapped a 32 Kb segment that ameliorated the 125 Kb plasmid’s carrying cost [6]. Here we report that deletion of this portion of plasmid pMB2 produces the smaller pRMKO plasmid that is sufficient to enhance autoaggregation in laboratory *E. coli* strains in a manner that pMB2 does not. We further demonstrate that the responsible locus was an orphan *papB/pefB* paralogue, without a cognate fimbrial cluster, and propose that this gene may optimize colonization and transmission in conjugation recipients.

The canonical, pathogenicity-island-encoded PapB regulator mediates crosstalk among different fimbriae in extraintestinal pathogenic *E. coli*, leveraging type I fimbrial phase variation to ensure that type I fimbriae and pyelonephritis-associated (P)-fimbriae are not expressed simultaneously [7, 8]. Type I fimbriae or *fim* genes are present in most *E. coli* whilst the P-fimbriae-encoding *pap* genes are typically only found in uropathogenic and other extraintestinal *E. coli*. ON to OFF phase variation of type I fimbriae is mediated by FimE recombination at the *fimS* switch locus within the *fim* cluster. FimB, an analogous site-specific recombinase, predominantly mediates the reverse *fimS* phase OFF to phase ON switching [9]. FimB switching behavior is repressed by PapB and PapB also promotes *fimE* expression, thereby shutting type I fimbriae OFF. Although *papB* lies within the chromosomal pyelonephritis-associated pilus (*pap*) cluster PapB controls *fim* expression even when expressed in *trans* from a plasmid in the laboratory [8, 10].

A number of PapB paralogues (PFAM03333) have been described in enteric bacteria, with varying *fim* regulatory activities. The family includes DaaA of the F1845 fimbrial cluster, SfaB of S-fimbriae, ClpB of CS31 fimbriae, FaeB of K88 fimbriae, FanA and FanB of K99 fimbriae, AfaA of the *afa* adhesin cluster as well as PefB of *Salmonella pef* fimbrial cluster [8, 11]. All these genes occur in the context of an adhesin gene cluster that they regulate and have the potential to crosstalk to other fimbriae.

## Main text

### Methods

#### Strains and plasmids

Bacterial strains (Table 1) were cultured in in Luria broth containing chloramphenicol (30 μg/mL), ampicillin (100 μg/mL), tetracycline (25 μg/mL), or neomycin (50 μg/mL), where required for selection, and maintained at − 70 °C in Luria broth:glycerol 1:1. Plasmid pMB2, originally isolated from an *Escherichia* isolate in Nigeria [6], and its derivatives are described in Table 1.

**Table 1:** Strains and plasmids used in this study

Designation	Description	Reference or source
Strains		
M63c	*E. coli* wt, S^R^, Nal^R^, Sul^R^, Amp^R^, Cip^R^, Te^R^, W^R^, K^R^	[20]
DH5α	F^–^ ø80d*lacZ*ΔM15 Δ(*lacZYA-argF*)U169 *deo*R *recA*1 *endA*1 *hsdR*17(rK^–^ mK^+^) *phoA supE*44 λ–*thi*-1 *gyrA*96 *relA*1	Invitrogen
ORN172	*thr-l leuB thi-1 A(argF-lac)U169 maL1l xyl-7 ara-13 26 mtl-2 gal-6 rpsL tonA2 supE44 Δ(fimBEACDFGH);*	[24]
EC1502	Rifampicin-resistant, plasmid free *E. coli* strain	University of Bradford
Plasmids		
pMB2	125 Kb Naturally occurring *aac(6′)-Ib-cr*-bearing plasmid	[6]
pMB80-2	Naturally occurring conjugative plasmid from enteropathogenic *E. coli* strain	[25]
pRMKO	93 Kb Miniplasmid constructed by deleting a 32,331 bp *Not*1—*Xba*1 fragment from parental plasmid pMB2	[6]
pRMC	pBluescript II SK containing a 32,331 bp *Not*1—*Xba*1 fragment from pMB2 cloned	[6]
pBAD/Thio-TOPO	Arabinose inducible expression vector	Invitrogen
pBluescript II SK +	High copy number cloning vector	Agilent
pINKpefB	*papB/pefB* paralogue from pMB2 cloned into pBAD/Thio-TOPO	This study
pLMJ50	90 Kb aggregative adherence plasmid from enteroaggregative *E. coli* strain 60A marked with a *cat* cassette	[31]

#### General microbiology and molecular biology procedures

Standard molecular biology procedures were used [12]. pMB2, pRMKO and pRMC were extracted using the Qiagen® Large Construct Kit, as previously described [6]. All other plasmid extractions used the Qiagen® MiniPrep Kit. Plasmids were electroporated into *E. coli* strains using a Bio-Rad micropulser. The pMB2 *papB/pefB* analogue was amplified using the oligonucleotides 5′CACCACTCCCTCCCCCTATCCAA-3′ and 5′-CTCACGGTAGAAATATTTAAGAGC-3′ and then cloned into pBAD/Thio-TOPO. Overexpression of a protein of size consistent with PapB by 1, 2 and 4% arabinose, and repression by 1% glucose was verified by SDS PAGE according to the pBAD/Thio-TOPO manufacturer’s protocols (Additional file 1: Figure S1). In vitro conjugation was effected by solid mating on LB agar as described previously. The number of transconjugant colonies per donor colony-forming units was computed as the plasmid transfer efficiency [13]*.*

#### Sequence analysis

Sequence was viewed in Artemis [14] and BLAST [15] searches were performed via Artemis on the NCBI platform. Multiple alignments were performed using Clustal W on the EBI server [16] and in MEGA6 [17]. Maximum likelihood phylogenies were computed in MEGA6.

#### Autoaggregation

The settling assay of Hasman et al. [18], with previously detailed slight modifications [19], was used to quantify autoaggregation. Overnight cultures were prepared in test medium containing selective antibiotics and where necessary, gene expression was induced from the arabinose promoter in the pBAD/Thio-TOPO vector with 1% arabinose for 90 min before the start of the assay. At the assay start, cultures of each strain were adjusted to the same optical density at 600 nm (OD_600_). Eight mL of each adjusted culture was placed into paired tubes. One tube in each pair remained static and the other was lightly vortexed before each optical density measurement. The tubes were incubated without shaking at 37 °C. At designated time points, 0.5 mL was removed from within 2 cm of the surface of the culture, and the OD_600_ was measured, diluting the sample if required. OD_600_ supernatant measurements for different strains were compared using a t-test.

## Results and discussion

### A PapB/PefB paralogue on pMB2 interferes with autoaggregation

**Fig. 1:**
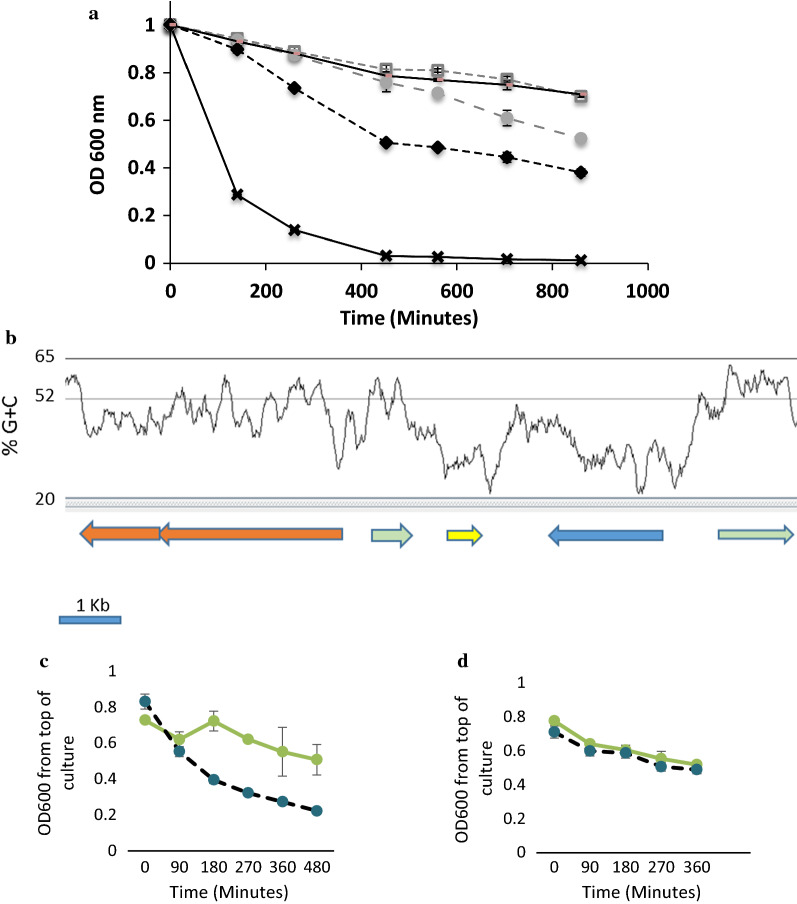
Autoaggregation in DMEM measured as absolute OD_600_ values from the top of a static culture sampled over time. **a** Autoaggregation of *E. coli* DH5α carrying pBluescript (grey closed circles on broken line), pMB2 (grey squares on broken line), pRMKO (black diamonds on broken line), pRMC (black line without marker) and control aggregative plasmid pLMJ50 (black crosses). Autoaggregation conferred by pLMJ50 (p < 0.001) and pRMKO (p < 0.01) is more pronounced than that conferred by pMB2 on DH5α. **b** The pefB region of pMB2. The pefB-like allele is colored yellow and is flanked by transposase/integrase genes (green). The *nik* transporter system genes are colored orange and the blue open-reading frame is a conserved hypothetical gene of unknown function. The %G + C content plot above the genes used a 120 nucleotide sliding window and the average value for the plasmid is 51.7%. **c** *E. coli* DH5α carrying pRMKO and pINKpefB1, the *pefB* gene from pMB2 cloned under the control of the arabinose promoter. Autoaggregation was measured after growth in arabinose (solid green line) or glucose (broken dark line). **d** *E. coli* ORN172 carrying pMB2 (solid green line) and pRKMO (dark broken line)

pMB2 was originally extracted from a commensal *Escherichia* isolate from Nigeria [20, 21]. In the course of functional analyses of fitness loci [6], we additionally observed that DH5α carrying the pRMKO miniplasmid autoaggregated significantly, particularly under low nutrient conditions. As shown in Fig. 1a, autoaggregation in liquid media was prominent in DH5α (pRMKO) but absent in DH5α (pMB2) cultures grown under the same conditions (p < 0.001). Cloning the 32 Kb deleted region into pBluescript also reduced autoaggregation of DH5α compared to an isogenic strain carrying the vector alone (Fig. 1a). We examined the sequence of the region deleted from pRMKO for genes with the potential to contribute to autoaggregation. The best candidate is small open reading frame (orf) located from 11,258–11,530 of the plasmid. The predicted amino acid sequence of this gene was 29% identical/50% similar to *E. coli* PapB from uropathogenic strain J96 (Genbank Accession ELL39276.1) and was 32% identical/ 48% similar to PefB, a PapB paralogue from *Salmonella enterica* serovariety Typhimurium strain LT2 (Genbank Accession AAL23523). There are no fimbrial genes proximal to the pMB2 *papB*-like gene, however there are mobility genes on either side and the *papB-*like gene region has a noticeably lower G + C content than the surrounding region (Fig. 1b).

We cloned the *papB/pefB* paralog under the control of the arabinose promoter and measured the clone’s ability to complement pRMKO hyperautoaggregation. As shown in Fig. 1c, when the *papB*/*pefB* paralogue is induced by arabinose, autoaggregation was diminished in DH5α (pRMKO, pINKPefB). This phenotype was not seen when the arabinose promoter is glucose-repressed (Fig. 1c). Based on this finding, we attribute the hyperautoaggregation phenotype conferred by pRMKO to deletion of the pMB2 *papB*/*pefB* paralogue.

### The pMB2 *papB*/*pefB* paralogue acts on chromosomally-encoded effectors

**Fig. 2:**
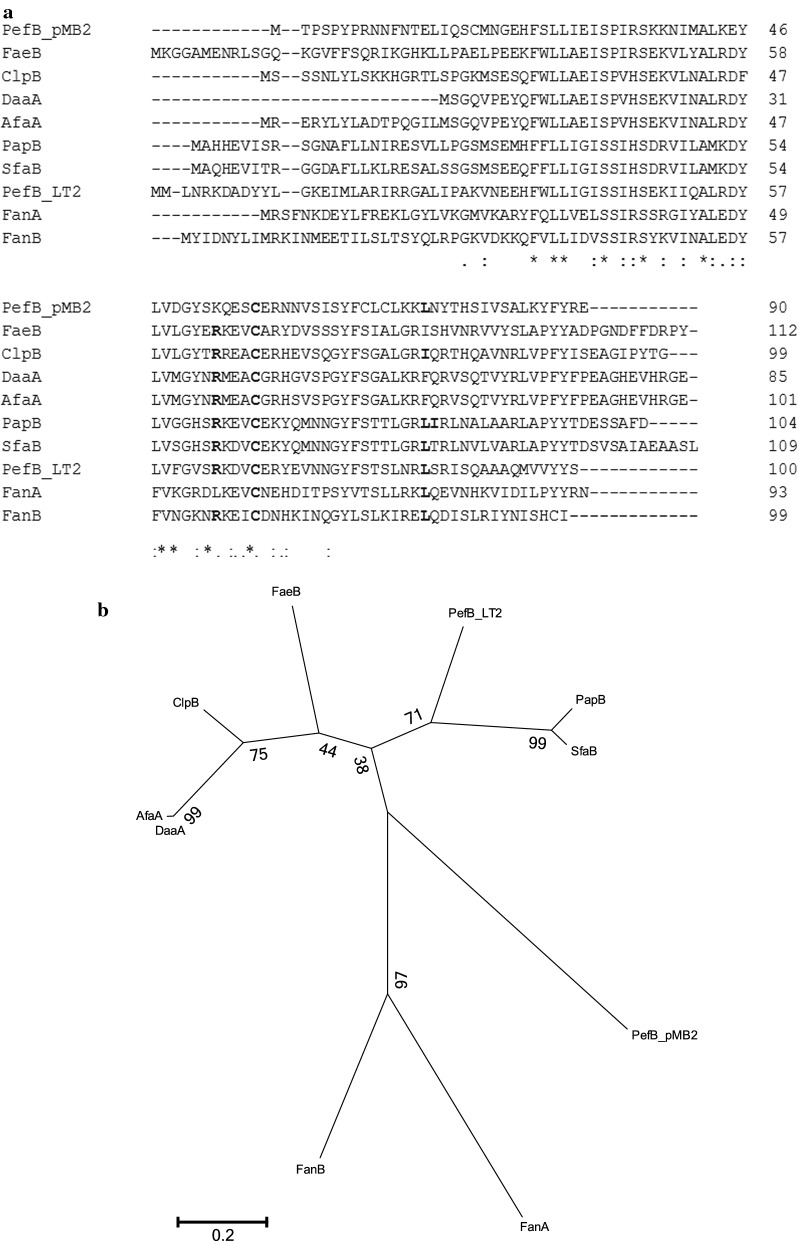
**a** Alignment of the pMB2-encoded PefB-like protein with a selection of known PapB homologs. PapB residues known to be required for fim-switching activity of PapB are indicated in bold. **b** Unrooted maximum likelihood tree of PapB paralogues including the PefB-like protein in this study (boxed), based on a Jones–Taylor–Thornton (JTT) substitution model. Bootstrap values from 1000 tree replicates generated using MEGA6 are given at branch points

PapB binds between *papI* and *papB* in the extraintestinal pathogenic *E. coli pap* cluster as well as, if less strongly, upstream of *fimE* [10]. R61A and C65A substitutions, within a hydrophilic region of PapB, produce derivatives unable to bind DNA [11]. As shown in Fig. 2, pMB2 PefB paralogue has a cysteine at the position equivalent to 65 in PapB. It does not have an arginine at position 61 but does have a positively-charged lysine residue at the equivalent position. Positions 86 and 91 at the PapB carboxy terminus are also essential for switching activity [8]. Holden et al. reported that L82F or F83Q substitutions, which altered these PapB residues to the equivalents in the non-binding DaaA paralogue, reduced switching. When both substitutions were made, activity was obliterated [8]. As shown in Fig. 2, the pMB2 PefB paralogue has a leucine at the position equivalent to PapB 82 but has an asparagine substitution at position 83, and so, even though it is considerably dissimilar to PapB, it could conceivably inhibit *fim* switching. However, Fig. 2 illustrates that there are many sequence differences between the allele we have identified and previously studied members of this family making it impossible to infer the nature and degree of its function or activity from sequence alone. In DH5α (pMB2), PapB/PefB could be repressing either chromosomally-encoded *fim* genes or pMB2 factors not deleted from pRMKO. From the complete sequence of pMB2, we did not identify genes encoding chaperone-user fimbriae or any other fimbrial type other than the F (conjugative)-pili, which are known to promote autoaggregation [6, 22, 23], although they lack a type I fimbriae-like switch. We additionally acknowledge that any of the several pMB2 orfs of unknown function could contribute to autoaggregation. To determine which possibility could be at play, we transformed pMB2 and pRMKO into ORN172, a *fim*-negative *E. coli* strain [24]. In this Δ*fim* background, there were no *papB*/*pefB*-associated differences in autoaggregation (Fig. 1d). Therefore, while it remains to be confirmed in direct experiments, this finding suggests that interactions of *papB*/*pefB* with core chromosomal factors, likely fimbriae, account for the hyperautoaggregation seen in DH5α (pRMKO) and that the hydrophobic residue at position 83 of PapB, may be less critical for FimB regulation than the presence of a leucine at position 82.

PapB is not known to directly regulate conjugative pili, but a *fim* repressor could conceivably enhance F-pilus-mediated autoaggregation and conjugation by preventing occlusion by chromosomally-encoded fimbriae. We performed an initial test of this hypothesis by comparing conjugative transfer rates from DH5α (pMB2) and DH5α (pRMKO) to strain EC1502. (The pRMKO miniplasmid contains the complete conjugative gene region of parental pMB2 plasmid). We recorded a transfer rate of 7.9 × 10^–6^ for DH5α (pMB2) in solid matings, compared to 3 × 10^–5^ of control plasmid pMB80 [25]. In contrast, conjugation from DH5α (pRMKO) could not be detected at the limits of the assay. Due to the low conjugation rate overall and the impossibility of detecting rates below 1 × 10^–8^ we could not perform a robust complementation experiment. However, altogether the available data support the idea that *papB*/*pefB* repressed genes could interfere with pMB2 conjugative transfer.

### The pMB2 *papB/pefB* paralogue is widely distributed and largely overlooked on extraintestinal *E. coli* plasmids

A BLAST search of the pMB2 nucleotide sequence range 7247–14,962, representing the *papB/ pefB* paralogue, its flanking low G + C region and the adjacent NikM/Hmu genes, reveal that it is a common plasmid-borne feature. Importantly, plasmids of *E. coli* O25b:H4-ST131 strains, commonly implicated in bloodstream, urinary and other extraintestinal infections, bear the entire region depicted in Fig. 1b. On the *E. coli* str. UMN026 plasmid p1ESCUM, often used an extraintestinal *E. coli* reference sequence (GenBank Accession Number: CU928148.1) [26], the entire region is present, but the *papB/pefB* paralog is not annotated as is the case for some well-known resistance plasmids [27], but not for others.

Cystitis and pyelonephritis isolates are more likely to contain one or more *pap* operons than other *E. coli*. Holden et al. [28], who observed this, also showed that cystitis and pyelonephritis isolates are better able to agglutinate red blood cells and attributed this to a higher copy-number and activity of intracellular PapB, which promoted expression of alternate fimbriae whilst shutting type I fimbriae OFF. *papB*/*pefB* paralogues on plasmids could achieve this effect irrespective of the presence of a full fimbrial operon consequently modulating the expression of core adhesins for in vivo adherence and/or exposure to the immune system. Thus, virulence factor profiling of pathogenic *E. coli* needs to take into account the available fimbrial genes as well as fimbrial regulators some of which—like the gene we characterize—could be plasmid-borne. As our *papB/pefB* paralogue is borne on a plasmid conferring resistance to no less than eight antimicrobial classes, antibiotic selective pressure could ultimately alter colonization and virulence profiles of strains in the wild.

In conclusion, pMB2 is self-transmissible by an F-type conjugative system that may function more effectively due to repression of chromosomal adhesins by a *papB*/*pefB* paralogue located on the same plasmid. Plasmids almost identical to pMB2 have been isolated from the USA (Accession number CP054458), Colombia (Acession number CP010372), France (Accession number LO017738), the UK (Accession number CP023372, CP018990) and elsewhere. The *papB*/*pefB* paralogue we describe is highly conserved among them and therefore the autoaggregation it represses is likely common, warranting further study. Our findings add to experimentally validated reports of two other non-resistance loci of pMB2 that promote *E. coli* survival and adaptability [6, 29]. Thus there are multiple genes resistance plasmids hat compensate the costs of their carriage or, as in this case, potentially propagate transmission. This study also provides yet another example of [30] horizontally acquired DNA that radically affects the expression of recipient cell core genes.

## Limitations

This study used convincing indirect methods to infer a regulatory link between the *papB/pefB* paralog on pMB2 and chromosomal adhesins and our findings agree with existing evidence on the mechanism of action of genes that belong to this family. We however lack the resources to demonstrate that the new *papB/pefB* paralog directly interacts with *cis* or *trans* fimbrial regulatory elements, or that fimbrial production is altered by the presence or absence of the *papB/pefB* gene.

## Supplementary Information

**Additional file 1: Figure S1**. Coomassie blue-stained SDS PAGE gel of whole cell lysates of DH5α pRMKO transformed with pINKpefB and grown in LB containing 0, 1, 2 and 4 % arabinose or 1% glucose showing induction of a protein of papB/pefB predicted size (7 KDa). Ladder: Prestained protein marker (Biorad).

## Data Availability

The sequence of plasmid pMB2, on which this manuscript is based is available from Genbank, Accession number MK492688. Other data generated or analysed during this study are included in this article.
