# Supplementary figures and images for: A plasmid-encoded papB paralogue modulates autoaggregation of Escherichia coli transconjugants
Source: BMC Res Notes. 2020 Dec 14;13:565. doi: 10.1186/s13104-020-05405-7 (PMC7734786; doi:10.1186/s13104-020-05405-7)

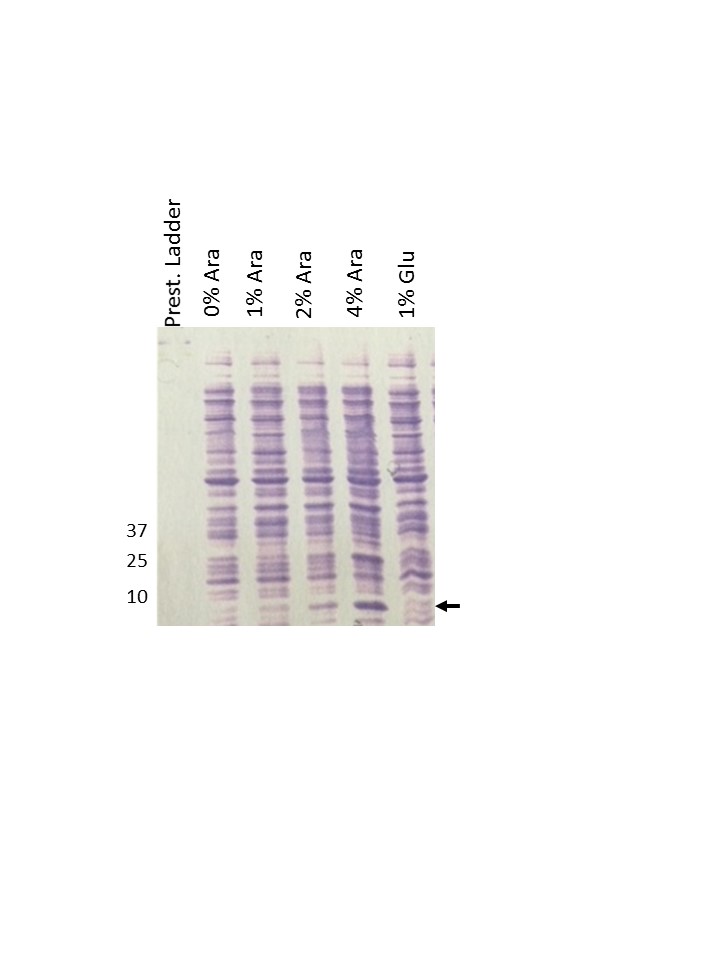

Supplement: Supplementary file 1 — Additional file 1: Figure S1. Coomassie blue-stained SDS PAGE gel of whole cell lysates of DH5α pRMKO transformed with pINKpefB and grown in LB containing 0, 1, 2 and 4 % arabinose or 1% glucose showing induction of a protein of papB/pefB predicted size (7 KDa). Ladder: Prestained protein marker (Biorad). [file 13104_2020_5405_MOESM1_ESM.jpg]
